# Baohuoside I Inhibits Tumor Angiogenesis in Multiple Myeloma *via* the Peroxisome Proliferator–Activated Receptor γ/Vascular Endothelial Growth Factor Signaling Pathway

**DOI:** 10.3389/fphar.2022.822082

**Published:** 2022-03-07

**Authors:** Ying Chen, Lina Zhang, Xiaoyan Zang, Xuxing Shen, Jianyong Li, Lijuan Chen

**Affiliations:** Department of Hematology, The First Affiliated Hospital of Nanjing Medical University, Jiangsu Province Hospital, Nanjing, China

**Keywords:** baohuoside I, PPARγ, multiple myeloma, angiogenesis, VEGF

## Abstract

Angiogenesis plays an important role in the development of multiple myeloma (MM). Baohuoside I (BI) is a core flavonoid monomer with anticancer property. However, the mechanism of BI on MM-stimulated angiogenesis has not been revealed. In this study, we demonstrated that BI inhibits MM-induced angiogenesis *in vitro* and angiogenesis in a xenograft mouse model *in vivo*. We further showed that peroxisome proliferator–activated receptor γ (PPARγ) transcriptional activity was mediated by a direct physical association between BI and PPARγ. Meanwhile, inhibition of PPARγ using lentivirus transfection of shRNA in human myeloma cell lines showed that the facilitation of PPARγ blocked angiogenesis and PPARγ repressed vascular endothelial growth factor (VEGF) transcription. Furthermore, BI treatment decreased VEGF expression, whereas VEGF expression remained unchanged after PPARγ knockdown when exposed to BI. Overall, our study is the first to reveal that BI inhibits MM angiogenesis by the PPARγ–VEGF signaling axis.

## Introduction

MM is an incurable disease characterized by malignant bone marrow (BM) plasma cell infiltration. Previous findings showed that greater BM angiogenesis was relevant with high risk of MM progression and adverse prognosis of patients. Lenalidomide, an immunomodulatory drug (IMiD), is widely used to treat patients with MM, whose mechanisms are multifactorial and include angiogenesis inhibition (Holstein and McCarthy 2017). Among the proangiogenic molecules, vascular endothelial growth factor (VEGF) plays an important role in maintaining angiogenesis; during the progression of myeloma, VEGFs trigger the angiogenic switch (Hong Wang et al., 2021; Zhiyao Wang et al., 2021) by stimulating vascular permeability and endothelial cell migration and proliferation. Moreover, VEGF has the ability to migrate epithelial cells from the microenvironment to the vicinity of the tumors and promotes the progression of cancers in some tumors, including lung adenocarcinoma (Jung et al., 2021), lymphoma (Shahini et al., 2017), and leukemia (Wang et al., 2015). Hence, antiangiogenic inhibitors block the VEGF pathway and have been widely used in the clinical treatment of tumors. Bevacizumab is a monoclonal antibody against VEGF, which has been successfully used as the first-line treatment for several cancers (Ferrara et al., 2005).

Epimedium is a tonic herbal drug which is extensively used as an anti-osteoporosis and cancer medicine in Chinese traditional medicine. It exerts antitumor effects through various mechanisms, including antiangiogenesis, apoptosis-inducing, cell cycle regulation, suppression of metastasis, and immunomodulation (Tan et al., 2016). Baohuoside I (BI), an important component of epimedium, has been confirmed to have antitumor effects in several studies. Guo et al. (2020) found that BI could inhibit hepatocellular carcinoma proliferation, migration, and invasion through targeting mTOR signaling. In human hepatocellular carcinoma cell lines, BI inhibited tumor proliferation by inducing apoptosis and reducing the NF-κB signaling pathway. It was also reported that BI suppressed metastasis and epithelial–mesenchymal transition of nasopharyngeal carcinoma by targeting the hedgehog pathway (Wang et al., 2021). However, there is little research about BI in MM. Kim et al. (2011) presented that BI inhibits multiple myeloma by inducing cell apoptosis. Nevertheless, the detailed molecular mechanisms were not clear.

In this study, we further explore the role of BI in MM angiogenesis both *in vivo* and *in vitro*. Simultaneously, we aimed to further identify the downstream targets and their regulatory interactions.

## Materials and Methods

### Cell Culture and Treatment

The human multiple myeloma cell lines (RPMI8226 and U266) were purchased from Cobioer Biosciences Co., Ltd. and grown in 1640 medium containing 10% or 20% FBS, 1% penicillin, and streptomycin. The human umbilical vein endothelial cells (HUVECs) were purchased from Cobioer Biosciences Co., Ltd. (Nanjing, China) and grown in HUVEC complete medium (Cobioer, Nanjing, China).

### MTT Assay

The cells (8 × 10^3^ cells) were cultured in medium containing 10% or 20% FBS and plated in 96-well plates for 48 h. The cells were then treated with different concentrations of BI for 48 h and treated with MTT-containing medium for 4 h. The supernatant was removed and then dimethylsulfoxide was added. The absorbance of the sample was measured at 570 nm using a spectrophotometer.

### Tube Formation Assay

To reconstitute the basement membrane, Matrigel (BD Biosciences, New Jersey, United States) was spread in a 96-well plate (50 μl/well) at 4°C and set aside for 30 min at 37°C. The HUVECs were cultured in a medium containing the MM cellular supernatant which were treated with various concentrations of BI and seeded on solidified Matrigel (1 × 10^4^ cells of each well). After incubation for 6 h, the cells were photographed to capture the capillary-like tubular structure.

### *In Vitro* Migration Assays

The cells (100 μl, 3 × 10^4^ cells) were resuspended in RPMI 1640 containing 1% FBS and grown in the upper chamber (Corning, New York, NY, United States). To the lower compartment was added 600 μl of the MM cell supernatant treated with BI. The migration chambers were cultured for 6 h under 5% CO_2_ at 37°C The lower invading cells were treated with 0.1% crystal violet and photographed. Calculation formula: (the treatment group/the control group) × 100%.

### Network Pharmacology

We used “multiple myeloma” and “BI” as the keywords to search the TCMSP database (https://old.tcmsp-e.com/tcmsp.php) and “multiple myeloma” in TTD (http://db.idrblab.net/ttd/), DrugBank (https://go.drugbank.com/), and OMIM databases (https://www.omim.org/) to predict the possible target of BI in MM. Then, using Cytoscape 3.7.1, we obtained the “drug–target–disease” network diagram of BI and analyzed it. As a result, PPARγ was preliminarily predicted to be the target of BI in MM. The affinity between the drug and target was detected using the PDB database (https://www.rcsb.org/) and AutoDock Grid. AutoDock Vina was used to simulate the molecular docking model of BI and drug disease target, and the molecular action mode was predicted. We then obtained a network diagram of BI and the corresponding disease targets using STRING and a diagram of the KEGG pathway when BI acted on MM using R and Perl to explore the possible signaling pathway *via* which BI functions in MM.

### Transfection of shRNA in MM Cells

The RPMI8226 or U266 cells were transfected with control shRNA or shRNAs targeting PPARγ using Lipofectamine 3000 reagent following the manufacturer’s protocol. The sequences of shPPARγ-1 and shPPARγ-2 are gga​tcc​GAC​AAA​TCA​CCA​TTC​GTT​ACT​CGA​GTA​ACG​AAT​GGT​GAT​TTG​TCT​TTT​TTg​aat​tc and gga​tcc​GCA​TTT​CTA​CTC​CAC​ATT​ACG​CTC​GAG​CGT​AAT​GTG​GAG​TAG​AAA​TGC​TTT​TTT​gaa​ttc, respectively. Cotransfection of the target gene plasmid and packing plasmid into 293T cells was carried out to generate lentivirus. The supernatant of the virus was obtained at 48 h. MM cells were sort-transfected using a flow cytometer (Beckman, CA, United States). Transduction efficiency was determined by Western blotting (WB).

### Cellular Thermal Shift Assay

General speaking, RPMI8226 cells were resuspended and incubated overnight. The cells were then exposed with 50 µM BI for 3 h and incremental temperatures (37, 47, 51, 53, 55, 57, 59, and 61°C) for 3 min. After treatment, the cells were placed in liquid nitrogen, thawed at room temperature, and vortexed briefly and pelleted by centrifugation. After determining the detection temperature, the cells were treated with BI (0, 0.4, 0.781, 1.563, 3.125, 6.25, 12.5, 25, and 50 µM) for 3 h. After collecting the cells, they were heated at 53°C for 3 min. They were then placed in liquid nitrogen, which was consistent with the abovementioned steps. After centrifugation, the supernatant was analyzed using WB.

### Western Blot Analysis

The cells were added to the microcentrifuge tube for collection. After extraction with RIPA, the protein concentration was determined using the Pierce BCA Protein Assay Kit. The protein was electrophoresed on a 10% SDS-PAGE gel and then transferred to a PVDF membrane (Bio-Rad). The membranes were immersed in a solution containing 5% non-fat dry milk and blocked for 1 h. Then, the PVDF membrane was incubated in primary antibody solution overnight at 4°C. The membrane was immersed in the corresponding secondary antibody. Finally, the proteins were visualized using the ECL kit. The primary antibodies are as follows: PPARγ (Cell Signaling Technology, Boston, MA, United States), VEGF (Proteintech, Chicago, IL, United States), and glyceraldehyde 3-phosphate dehydrogenase (GAPDH) (Proteintech, Chicago, IL, United States).

### Enzyme-Linked Immunosorbent Assay

VEGF expression was determined using a human VEGF ELISA kit (Fcmacs, Inc., Nanjing, China). RPMI 8226 and U266 cells were seeded at 8 × 10^3^ cells of each well and incubated for 48 h. After adding the stop solution, the OD450 nm was measured within 5 min.

### Reverse Transcription–Polymerase Chain Reaction

Cellular RNA was extracted using TRIzol (Invitrogen, CA, United States), and cDNA synthesis was performed *via* reversed transcription using Super Script II reverse transcriptase (Vazyme, Nanjing, China). qRT-PCR analysis of gene expression was performed using SYBR Green Master Mix (Vazyme, Nanjing, China). The expression level of target gene mRNA was calculated with the expression level of GAPDH as the reference. The following human primers were used in this study: GAPDH-F, GTCGGAGTCAACGGATT; GAPDH-R, AAGCTTCCCGTTCTCAG; PDK4-F, CCC​GCT​GTC​CAT​GAA​GCA​GC; PDK4-R, CCA​ATG​TGG​CTT​GGG​TTT​CC; CD36-F, ACA​GAT​GCA​GCC​TCA​TTT​CC; CD36-R, GCC​TTG​GAT​GGA​AGA​ACA​AA; VEGF-F, TAC​CTC​CAC​CAT​GCC​AAG​TGG​T; and VEGF-R, AGG​ACG​GCT​TGA​AGA​TGT​AC.

### Chromatin Immunoprecipitation

ChIP assays used the CHIP kit (Beyotime) for subsequent operations. The VEGF promoter primers were used in this study: VEGF1-F, CTGGCGGGTAGGTTTGA; VEGF1-R, GGA​AGA​GGA​CCT​GTT​GGA​G; VEGF2-F, GGAGCCTGCCAAGTGGT; VEGF2-R, CCA​TCG​GTA​TGG​TGT​CCT​AA; VEGF3-F, GGGTTGAGGGCGTTGGA; and VEGF3-R, GCATTGGCGAGGAGGGA.

### Luciferase Reporter Gene Assay

Chimeric firefly luciferase reporter plasmids were constructed as previously described. The human VEGF promoter region was subcloned into the pGL3-luciferase reporter plasmid (Genscript, Nanjing, China) at the NheI-HindIII site to generate the chimeric pGL3-basic_VEGFA-promoter reporter construct. RPMI8226 cells were plated in a 24-well dish. The plasmids were transfected using Lipofectamine 3000 reagent. After transfection for 6 h using Lipofectamine 3000, the cells were exposed to BI, RSG, or GW9662-containing medium for 24 h. Then, the kit (Promega, Madison, WI, United States) was used for subsequent operations, and finally a microplate reader was used for detection.

### Human Myeloma Xenograft Mice Model

RPMI8226 cells (5 × 10^6^) were subcutaneously injected into the abdominal area of 6∼8- week-old BALB/c nude mice (*n* = 6 per group). After 3 days of subcutaneous injection, the mice were injected with BI (25 mg/kg) every other day. The size of the tumor was regularly measured. The mice were killed when tumors reached 20 mm in diameter. The formula (length × width^2^)/2 was used to calculate tumor volume (Wang et al., 2021).

### Immunohistochemistry

The methanol-fixed tissues were embedded in paraffin and cut into 4- to 5-µm slices. The tissue sections were deparaffinized and dehydrated. The slides were exposed to in 10 mM citrate buffer (pH 6.0) for 10 min in a microwave to induce antigen unmasking. The slides were added with anti-VEGF (Proteintech, Chicago, United States) and CD34 (Invitrogen, CA, United States) antibodies at 4°C overnight and used for secondary antibody incubation the next day for 1 h at room temperature. After developing the chromogen, hematoxylin was used for counterstaining.

### Data Analysis and Statistical Methods

GraphPad Prism software (GraphPad Software Inc.) was used to process the data and plot. Two experimental groups were analyzed using Student’s t-test. The differences between the groups were set at *p < 0.05, **p < 0.01, and ***p < 0.001.

## Results

### Baohuoside I Inhibits MM Angiogenesis *In Vitro*

**FIGURE 1:**
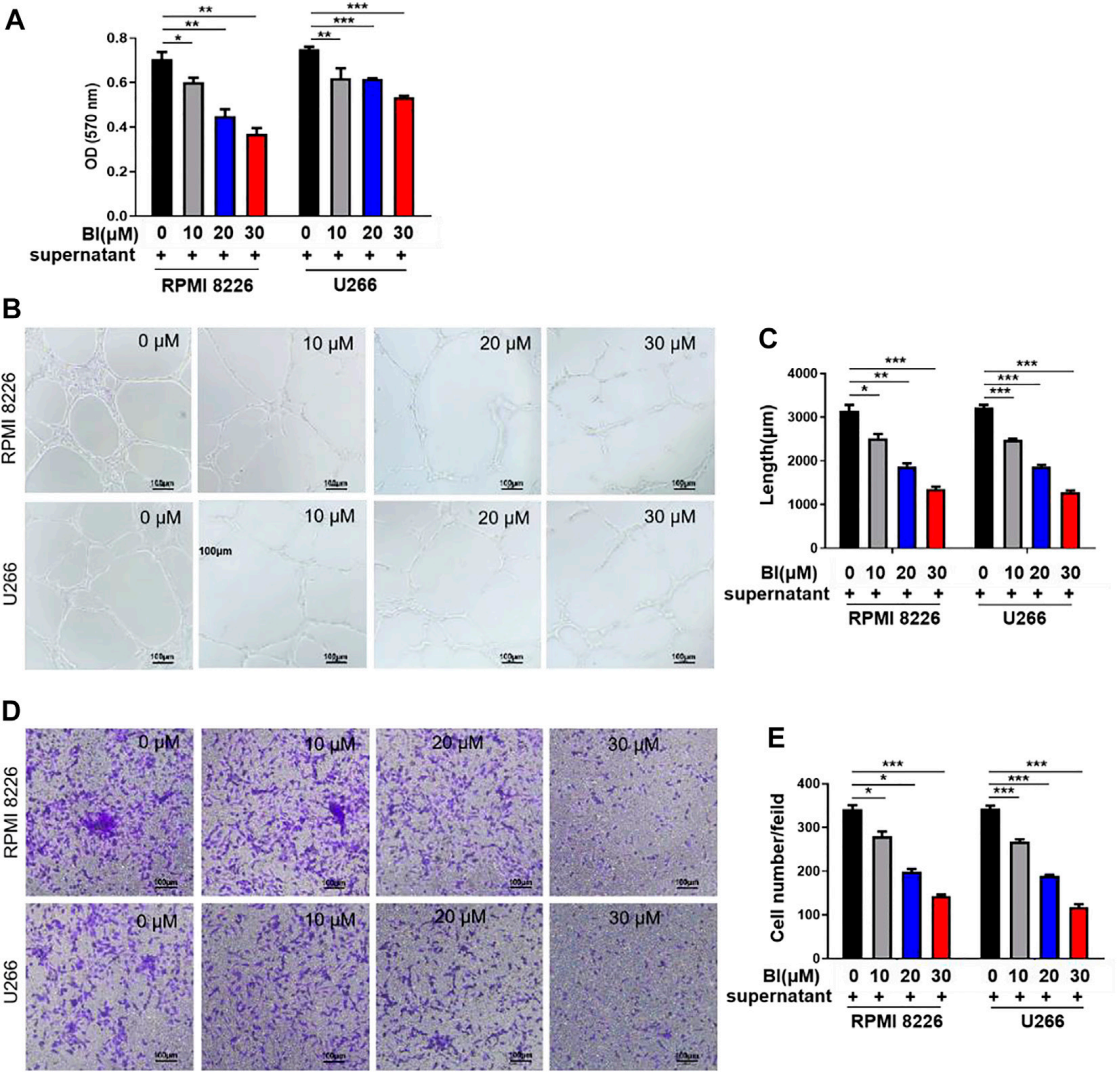
Baohuoside I (BI) inhibited multiple myeloma (MM)–induced angiogenesis. **(A)** BI-treated MM cell supernatant inhibits the HUVECs. The value of optical density (OD) was determined by MTT assay at an absorbance of 570 nm. **(B,C)** Tube formation experiment was carried out by adding HUVECs and BI-treated MM cell supernatant to the matrix gel. **(D,E)** HUVEC migration experiment evaluates the migration ability of cells through the transwell chamber.

We investigated the effects of BI on HUVECs using BI-treated MM cell supernatants. As shown in Figure 1A, BI-treated MM cell supernatants significantly reduced the growth of HUVECs (0 μM BI-treated supernatant vs. 10 μM BI treated supernatant in 8,226: 0.71 ± 0.05 vs. 0.60 ± 0.04, *p* < 0.05; 0 μM BI-treated supernatant vs. 10 μM BI-treated supernatant in U266: 0.75 ± 0.02 vs. 0.62 ± 0.08, *p* < 0.01; Figure 1A). To estimate the effect of BI-treated MM cell supernatants on tubular formation of endothelial cells, we performed a HUVEC tube formation experiment and revealed that with the increase of BI concentration, BI treatment significantly inhibited the tubule structure formation (Figures 1B,C). Furthermore, the effects of the BI-treated MM supernatant on migration and invasion were estimated using the HUVEC transwell assay. The result showed that the BI-treated MM supernatant inhibited migration and invasion in a concentration-dependent manner (Figures 1D,E). The results imply that BI suppresses angiogenesis in MM *in vitro*.

### Baohuoside I Inhibits MM Angiogenesis *In Vivo*

**FIGURE 2:**
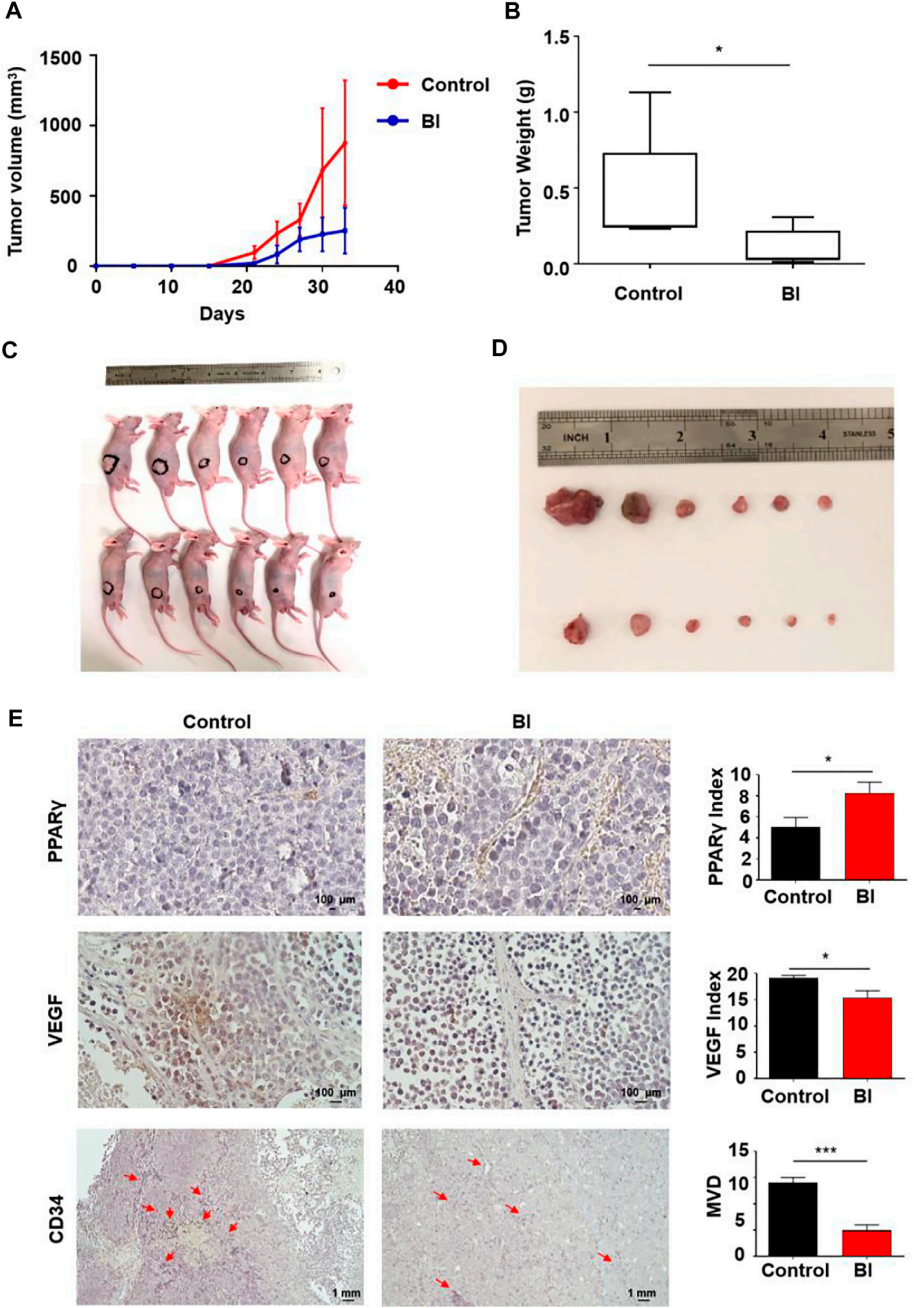
Anti-MM angiogenesis of BI in an MM xenograft mouse model. **(A–D)** Mice injected subcutaneously with RPMI8226 cells. After injection with control or BI, the tumor volume was recorded. **(E)** Immunohistochemistry labeled PPARγ, VEGF, and CD34 in mouse xenograft tumors.

In view of the inhibitory effect of BI on angiogenesis *in vitro*, we explored its impact on MM angiogenesis *in vivo*. The mice were injected with RPMI 8226 cells subcutaneously. Tumor lumps in BI-treated mice developed slower than those in the control (control vs. BI at day 33: 875.78 ± 444.84 mm^3^ vs. 251.64 ± 162.41 mm^3^; *p* < 0.01; Figure 2A). At 33 days, the neoplasm weights of the mice in each group were measured. We observed that the BI treatment xenograft tumor group was lighter than the control group (control vs. BI: 0.45 ± 0.36 g vs. 0.10 ± 0.12 g, *p* < 0.05; Figures 2B–D). The immunohistochemistry assay revealed that the PPARγ expression in tumor tissue with the BI injection group was increased (control vs. BI: 5.06 ± 0.90 vs. 8.28 ± 1.02, *p* < 0.05). VEGF expression in the tumors of the BI treatment group was reduced (control vs. BI: 19.09 ± 0.53 vs. 15.42 ± 1.30, *p* < 0.05), and the microvessel density (MVD) of CD34-labeled tumor tissue in the BI injection group was significantly lower than than that of the control group (control vs BI: 14.00 ± 1.00 vs. 5.00 ± 1.00, *p* < 0.001; Figure 2E).

### Baohuoside I Induces Peroxisome Proliferator–Activated Receptor γ Activation

**FIGURE 3:**
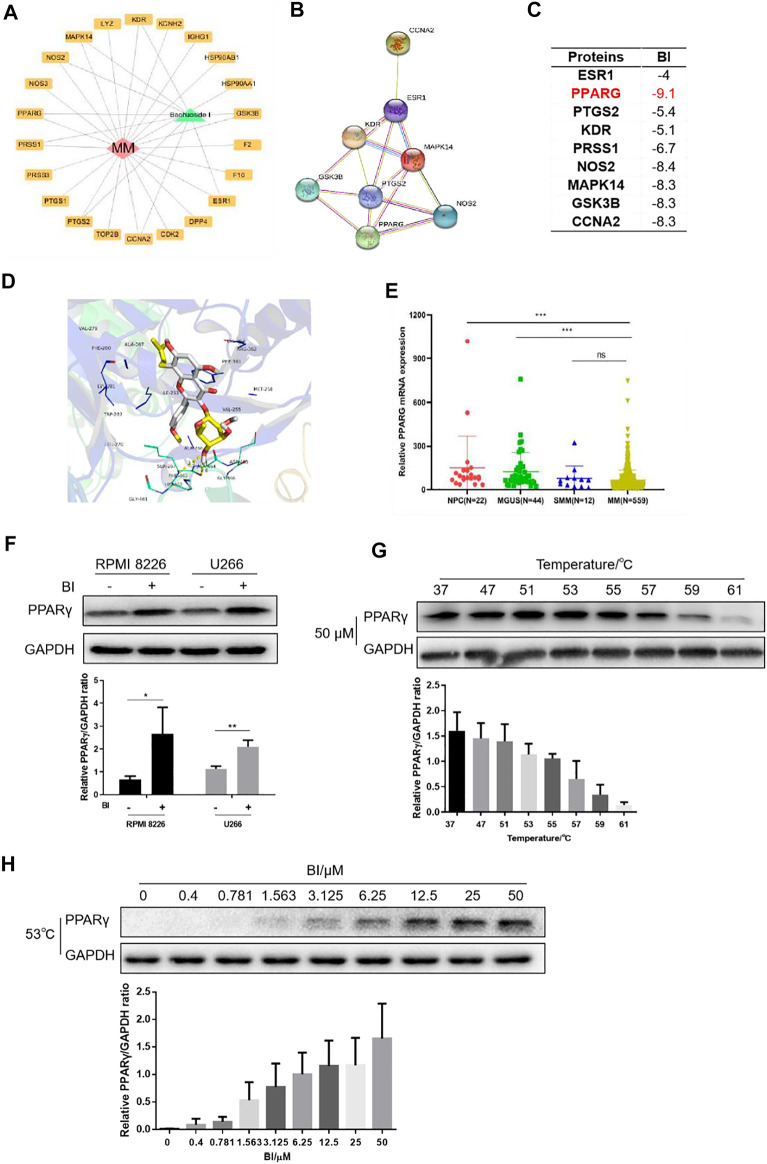
BI displays a binding ability to PPARγ. **(A)** Cytoscape was used to draw BI’s “drug–target–disease” network diagram with MM. **(B)** Interaction between protein and protein is obtained through STRING (https://www.string-db.org/). **(C)** Affinity between BI and targets was detected through the PDB database and AutoDock Grid. **(D)** Molecular docking model of BI and the drug disease target was simulated by AutoDock Vina. **(E)** Expression of PPARγ mRNA in normal people and in MGUS, SMM, and MM patients was examined by the GEO database (GSE5900 and GSE2658). **(F–H)** Protein expression of PPARγ was determined by WB after MM cells were exposed to BI **(F)**; after MM cells were heated by incremental temperature at 50 μM BI-treatment **(G)**; and after MM cells were exposed with increased concentration of BI at 53°C **(H)**.

To identify the relevant targets of BI, nine targets corresponding to the ingredient of BI were screened in the STITCH (http://stitch.embl.de/), PubChem (http://pubchem.ncbi.nih.gov/) and ChEMBL (http://www.ebi.ac.uk/chembl/) databases. These target genes included the estrogen receptor (*ESR1*), peroxisome proliferator–activated receptor gamma (*PPARG*), vascular endothelial growth factor receptor 2 (*KDR*), prostaglandin G/H synthase 2 (*PTGS2*), glycogen synthase kinase-3 beta (*GSK3B*), trypsin-1 (*PRSS1*), nitric oxide synthase (*NOS2*), mitogen-activated protein kinase 14 (*MAPK14*), and cyclin-A2 (*CCNA2*) (Figure 3A); the interactions are shown in Figure 3B. A lower binding energy between the ligand and receptor indicates higher likelihood of binding with each other. The molecular docking affinity of BI with the target protein PPARγ was far less than that of the others, indicating that BI had good binding activity with PPARγ. Furthermore, we simulated the molecular structure of BI and PPARγ interaction. In Figure 3D, the hydroxy group of BI formed a hydrogen bond with Phe 463 and Ala 464 of PPARγ, and the BI benzene ring could interact with Phe 361 of PPARγ *via* the π–π conjugate. Intriguingly, PPARγ expression evaluated by GEO datasets (GSE2658 and GSE5900) showed significant downregulation of PPARγ in 559 MM patients compared with those in the NPC (normal people) and MGUS patients (Figure 3E). We tested the effects of BI on PPARγ protein expression. When cells were treated with BI, the PPARγ protein expression increased (Figure 3F). Furthermore, we used a cellular thermal shift assay to validate the database predictions. The experimental results showed that PPARγ was gradually degraded with increasing temperature when treated with 50 μM BI. The protein could be easily detected at 53, 55, and 57°C. When it reached 61°C, PPARγ was almost completely degraded (Figure 3G). Therefore, we chose 53°C to investigate the degradation of PPARγ with the increase of BI concentration. The experimental results showed that as the BI increased, PPARγ was less likely to degrade (Figure 3H). These results suggest that BI displays a binding ability to PPARγ.

### Peroxisome Proliferator–Activated Receptor γ Inhibits Angiogenesis

**FIGURE 4:**
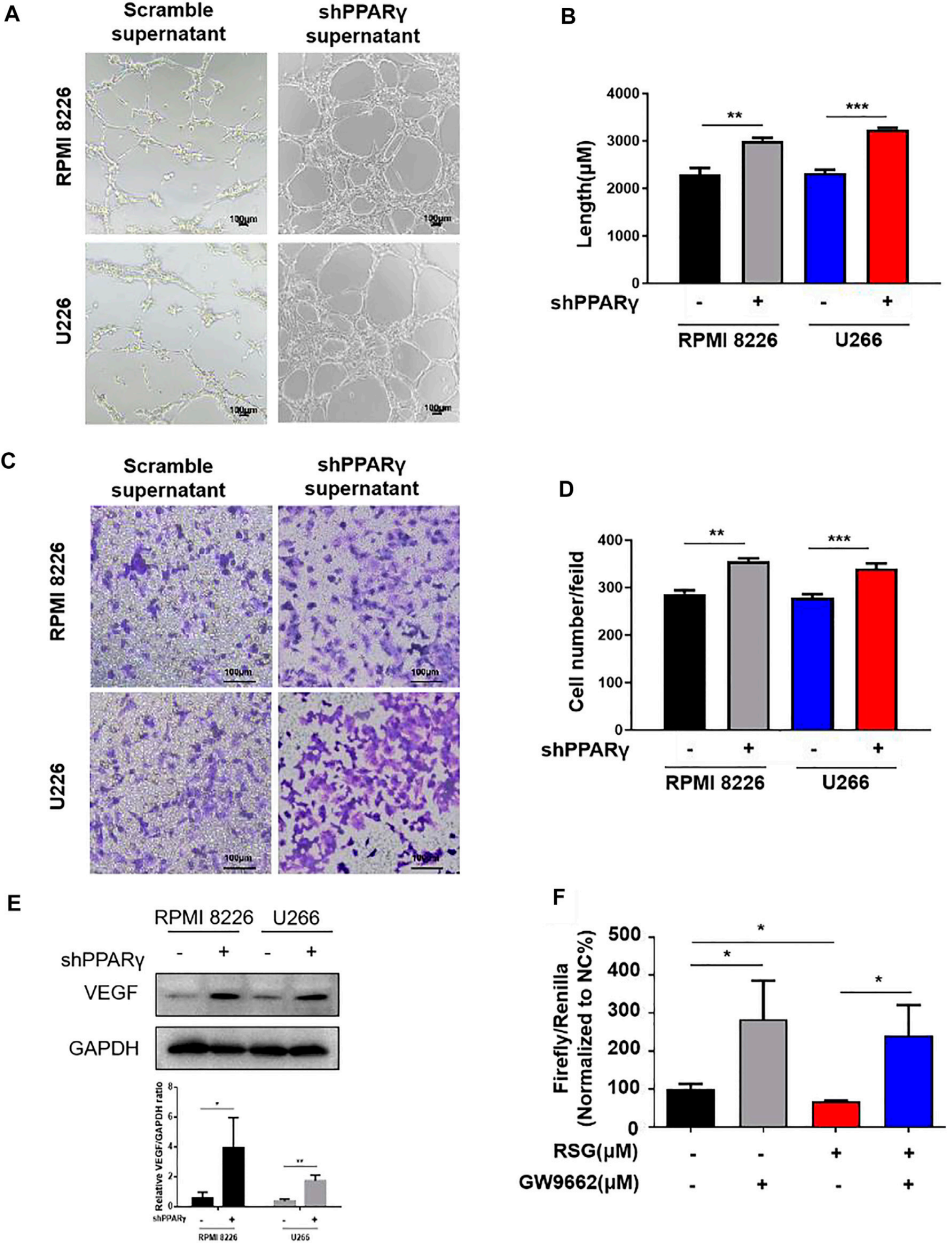
PPARγ knockdown MM cells promote MM-induced angiogenesis. **(A–D)** Tube formation experiment and HUVEC migration experiment were treated by the MM supernatant with or without PPARγ knockdown. **(E)** Protein level of VEGF was measured by WB at MM cell PPARγ-knockdown or not. **(F)** Luciferase reporter gene experiment detects the transcriptional activity of PPARγ when treated with agonist (RSG) and antagonist (GW9662).

We investigated the effect of PPARγ on HUVECs using the MM cell supernatant. We constructed a PPARγ knockdown MM cell line (Supplementary Figure S1A). Compared to the scramble group, the supernatants from PPARγ-knockdown MM cell lines promoted HUVEC tube formation (Figures 4A,B). The same tendency was also observed in HUVEC migration assay (Figures 4C,D). Furthermore, the supernatants of the PPARγ agonist rosiglitazone (RSG) suppressed HUVEC tube formation and migration, but the inhibition effect could be reversed by PPARγ antagonists (Supplementary Figures S1B,C). The detection of proteins extracted from the MM cells showed that VEGF expression was upregulated after PPARγ knockdown (Figure 4E). Moreover, we revealed the effect of PPARγ on VEGF transcription. Luciferase reporter gene experimental results showed that RSG suppressed VEGF transcription; the PPARγ antagonist GW9662 could promote VEGF transcription, and the inhibition of VEGF transcription with RSG treatment was reversed by the addition of GW9662 (Figure 4F). Overall, PPARγ inhibited angiogenesis probably by suppressing VEGF transcription.

### Baohuoside I Blocks Angiogenesis Through the Peroxisome Proliferator–Activated Receptor γ-Vascular Endothelial Growth Factor Pathway

**FIGURE 5:**
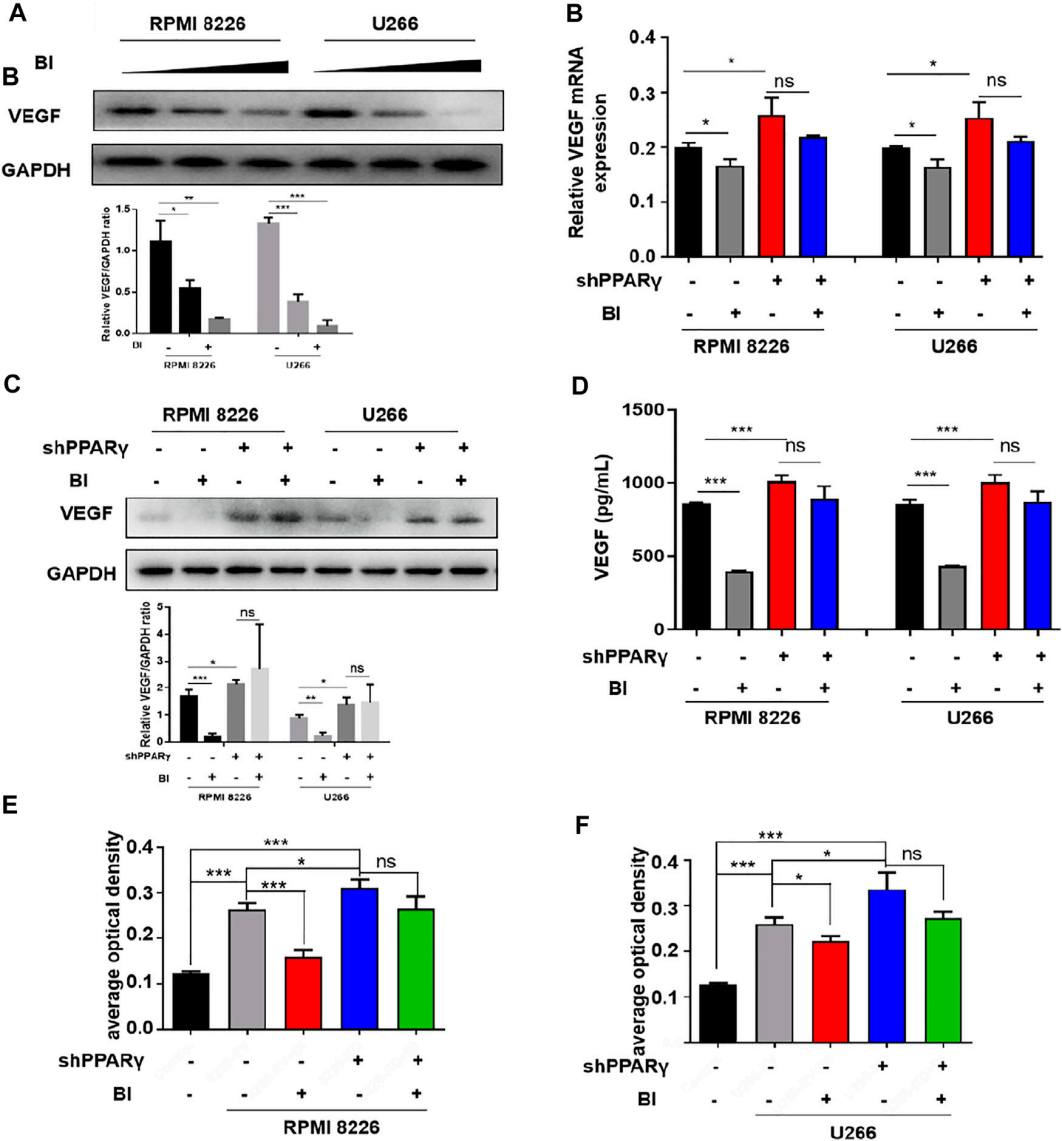
Inhibition of VEGF by BI requires PPARγ. **(A)** VEGF expression in MM cells was determined by WB after BI treatment. **(B,C)** mRNA and protein expression of BI-treated MM cells after PPARγ knockdown were detected by WB and PCR, respectively. **(D)** Expression of VEGF in the supernatant of MM cells was detected by ELISA. **(E,F)** Growth of HUVECs exposed at the MM cell supernatant which was in the presence or absence of BI and PPARγ knockdown and detected by MTT.

Given that BI can inhibit angiogenesis and bind to PPARγ, we further explored the relationship between BI, PPARγ, and VEGF. The experimental results showed that VEGF expression gradually decreased as BI concentration increased (Figure 5A). Following the knockdown of PPARγ and BI treatment of MM cells, VEGF protein and mRNA expression did not decrease significantly, in either RPMI8226 or U266 cells (Figures 5B,C). Immediately afterward, VEGF expression in the MM cell supernatant was detected. The ELISA assay showed that after BI administration, VEGF in MM cell supernatants was significantly downregulated; however, the same result was not observed in MM cells after PPARγ knockdown (Figure 5D). After treatment with the MM cell supernatant, the growth of HUVECs was significantly increased. However, after the administration of the BI-treated MM cell supernatant, this pro-proliferative effect was inhibited. When HUVECs were exposed to the BI-treated MM supernatant, compared with the supernatant of PPARγ non-knockdown cells, the supernatant of PPARγ knockdown cells exerted a nonsignificant effect on HUVEC proliferation (Figures 5E,F). In summary, BI inhibits VEGF expression through PPARγ.

**FIGURE 6:**
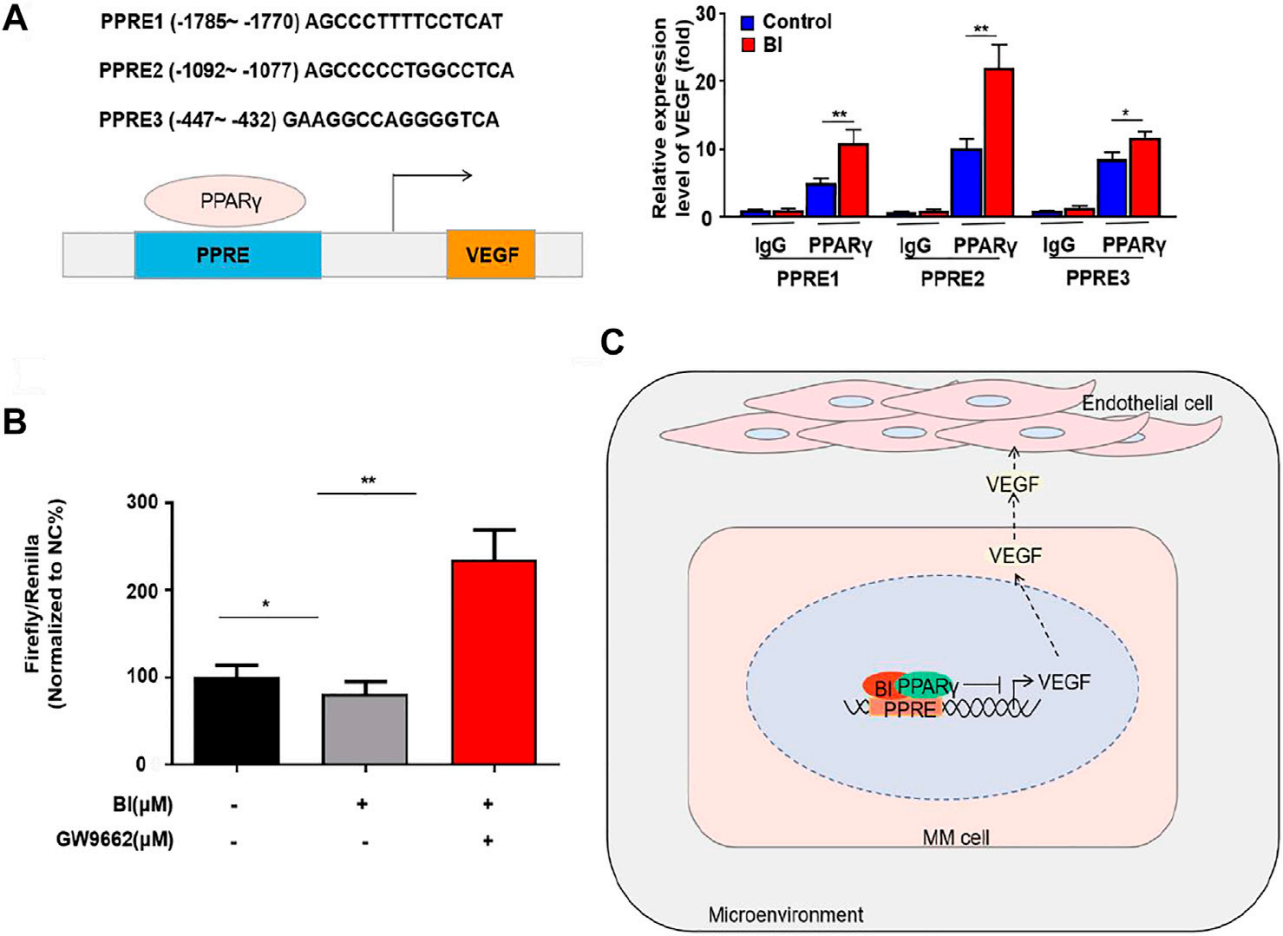
BI enhanced the activation of PPARγ. **(A)** ChIP assay detects the binding site of PPARγ in the VEGF promoter region. **(B)** Luciferase reporter gene experiment detects the effect of BI and GW9662 on the transcriptional activity of PPARγ. **(C)** Schematic model of the underlying mechanism of BI in MM. Abbreviations: BI, baohuoside I; PPARγ, peroxisome proliferator–activated receptor γ; PPRE, receptor responsive element; VEGF, vascular endothelial growth factor.

To explore the specific binding position of PPARγ to the VEGF promoter, we conducted ChIP experiments which showed that PPARγ could bind to PPRE1 (AGCCCTTTTCCTCAT), PPRE2 (AGCCCCCTGGCCTCA), and PPRE3 (GAAGGCCAG GGGTCA). This binding ability was enhanced after BI treatment of MM cells (Figure 6A). Furthermore, the luciferase reporter gene assay revealed that the relative luciferase activity of MM cells was obviously decreased after BI treatment. In addition, this inhibition was reversed by the combination of GW9662 (Figure 6B). The abovementioned results indicated that BI could inhibit VEGF by enhancing PPARγ activation.

## Discussion

In the recent 2 decades, with the successful application of immunomodulatory drugs, proteasome inhibitors, monoclonal antibodies, and CART cell therapies in MM treatment, the prognosis of MM patients has been greatly improved. However, MM remains incurable because nearly all the patients are subject to drug resistance and relapse (Rajkumar and Kumar 2020). Therefore, it is urgent to explore new therapeutic targets and develop novel drugs. Ria et al. (2014) demonstrated that downregulation of HIF-1α in MM BM endothelial cells could inhibit angiogenesis and restore the sensitivity of bortezomib and lenalidomide. The results of this study highlighted a novel strategy to treat myeloma by inhibiting neovascularization.

Traditional Chinese medicine is regarded as a treasure house for developing new drugs. Recently, studies confirmed that Chinese herbs could block angiogenesis and cause the death of MM cells. *In vivo* and *In vitro* studies showed that wogonin (an active ingredient in *Astragalus*) repressed MM-stimulated angiogenesis through the c-Myc-VHL-HIF-1α signaling pathway (Fu et al., 2016). Chen et al. (2010) revealed that artesunate (an agent extracted from *Artemisia annua*) could significantly reduce the level of Ang-1 and VEGF secreted by myeloma cells to cause the reduction of angiogenesis. Epimedium is one of the classic traditional Chinese medicines commonly used to treat MM. Epimedium tonifies the kidney and strengthens the bone. Coincidentally, MM is often accompanied by renal insufficiency and bone destruction. Research has shown that the common metabolite of epimedium flavonoids (e.g., icariin, epimedin A, sagittatoside A, epimedin B, and sagittatoside B) is BI (Zhong et al., 2019). In addition to its bone-strengthening effects, BI has been widely studied for its anticancer role in recent years. Our study describes the mechanism of BI acting on MM (Figure 6C). We first explored the effect of BI on myeloma and revealed that it has an obviously inhibiting effect on angiogenesis. Moreover, we explored that the main target of BI is PPARγ.

Peroxisome proliferator–activated receptor γ (PPARγ) is a member of the nuclear hormone receptor superfamily and a ligand-activated transcription factor that participates in differentiation, proliferation, and tumorigenesis in cells by regulating the expression of target genes (Liu et al., 2020). This study shows that BI was a potential agonist of PPARγ. BI showed binding activity to PPARγ both theoretically and practically. We predicted that BI showed a theoretical possibility of hydrogen bonding with Phe 463 and Ala 464 and the π–π action with Phe 361 using databases and software. BI showed a similar effect to that of the PPARγ agonist and could be competitively bound by GW9662. Once BI binds to PPARγ, PPARγ is activated, and downstream target genes are regulated. These results are consistent with those of the current research; for example, isorhamnetin decreases the growth of gastric cancer *via* activating PPARγ (Ramachandran et al., 2012); Epigallocatechin-3-O-gallate *via* activation of PPARγ upregulates Pim-1 to protect the vascular (Liu et al., 2013); and morin attenuate synovial angiogenesis by activation of PPARγ (Yue et al., 2018). Above all, this study showed here that BI could bind to and activate PPARγ in MM, so it is a potential agonist of PPARγ.

Moreover, we revealed that BI significantly inhibited MM-stimulated angiogenesis. Angiogenesis was obviously inhibited *in vitro* when exposed in the supernatant from BI-treated MM cells. Consistently, BI inhibited the expression of VEGF and lowered MVD in xenograft tumors. In MM cells, BI also decreased the levels of VEGF, the main secretory cytokine in MM-stimulated angiogenesis. Studies have shown that PPARγ can inhibit VEGF transcription-induced tumor angiogenesis by binding to the receptor responsive element (PPRE) in the VEGF promoter region *in vitro*, decrease the VEGF receptor expression in vascular endothelial cells, and suppress tumor angiogenesis (Wagner and Wagner 2020). However, the molecular crosstalk between PPARγ and neovascularization in MM has never been investigated to date. In this study, in PPARγ-knockdown MM cell medium cultured with HUVECs, the tube formation and migration were significantly inhibited. We subsequently found that PPARγ knockdown induced an increase in the VEGF protein level. In addition, MM-secreted VEGF was significantly elevated following PPARγ knockdown. To further explore whether PPARγ was directly involved in VEGF transcription in MM cells in a previous study (Fang et al., 2016), we used the PPARγ ligand agonist RSG and antagonist GW9662 to detect its effect on transcriptional activation (Schubert et al., 2020; Fu et al., 2021). The experimental results showed that VEGF transcription was increased after PPARγ inhibition and vice versa. Moreover, we also detected the binding site of PPARγ on VEGF, such as PPRE1-1785 AGC​CCT​TTT​CCT​CAT-1770, PPRE2-1092 AGC​CCC​CTG​GCC​TCA-1077, and PPRE3-447 GAA​GGC​CAG​GGG​TCA-432.

Finally, we determined whether BI affected MM-stimulated angiogenesis *via* the PPARγ/VEGF axis. Our results showed that in PPARγ-knockdown MM cells, BI could not inhibit the mRNA and protein levels of VEGF or the secreted VEGF level. Furthermore, BI reduced the growth of HUVECs, whereas in PPARγ-knockdown MM cells, it lost its effect. GW9662 treatment almost completely reversed the downregulation of VEGF transcription in MM cells with BI treatment.

In conclusion, this study discovers that BI inhibited MM-stimulated angiogenesis *via* the PPARγ/VEGF axis. We believe BI is a promising drug for the treatment of MM.

## Data Availability

The datasets presented in this study can be found in online repositories. The names of the repository/repositories and accession number can be found below: DRYAD, DOI: 10.5061/dryad.79cnp5hxf.

## Supplementary Material

Supplementary Figure 1 PPARγ knockdown MM cells promote MM-induced angiogenesis. **(A)** Expression of PPARγ was determined by WB in MM cell transfection with lentiviruses encoding the shPPARγ and vector. **(A,B)** Tube formation experiment and HUVEC migration experiment were performed by analyzing the effects of the MM cell supernatant on the tube formation and migration of HUVECs treated with RSG or GW9662.
