# Supplementary figures and images for: Baohuoside I Inhibits Tumor Angiogenesis in Multiple Myeloma via the Peroxisome Proliferator–Activated Receptor γ/Vascular Endothelial Growth Factor Signaling Pathway
Source: Front Pharmacol. 2022 Mar 7;13:822082. doi: 10.3389/fphar.2022.822082 (PMC8948427; doi:10.3389/fphar.2022.822082)

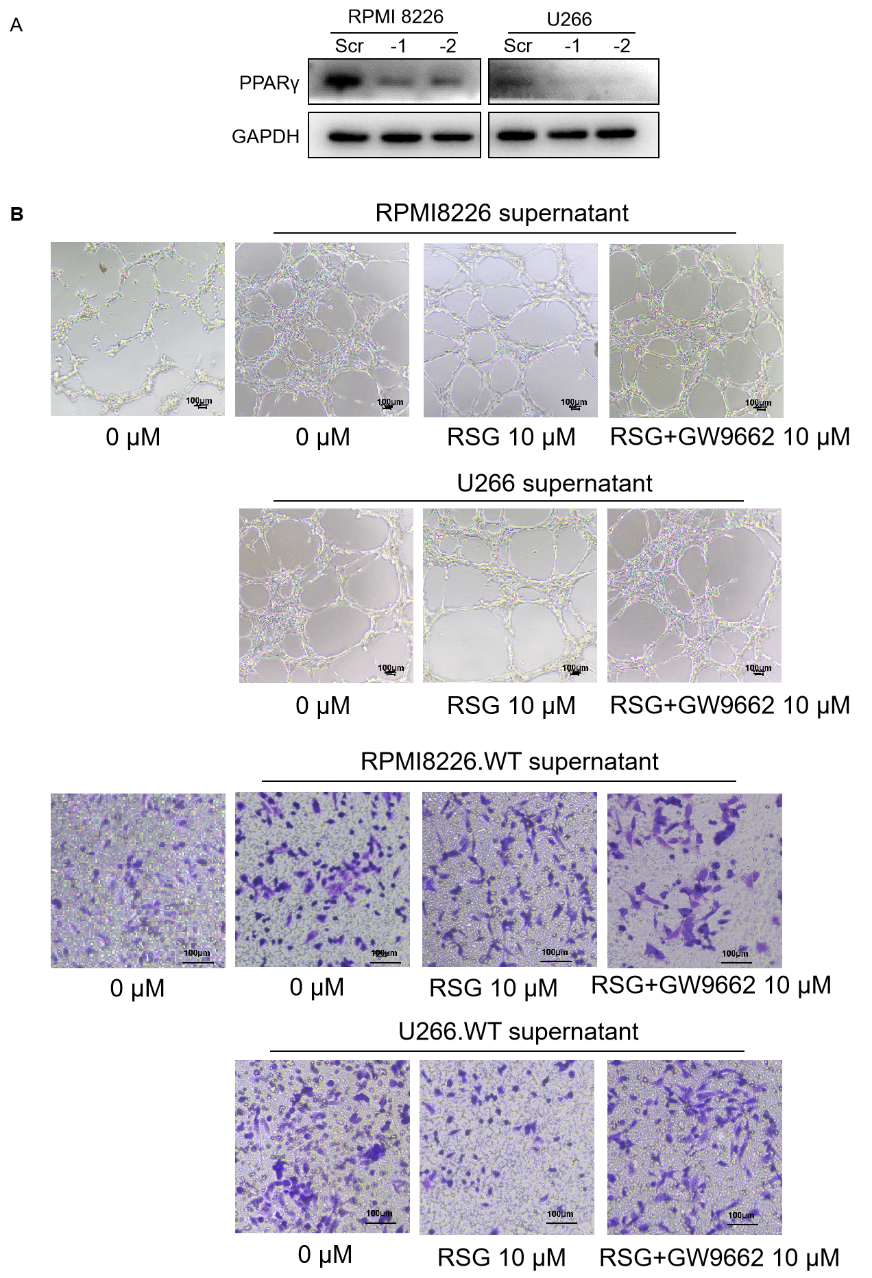

Supplement: Supplementary file 1 [file Image1.JPEG]
